# The response of grain yield and ear differentiation related traits to nitrogen levels in maize varieties with different nitrogen efficiency

**DOI:** 10.1038/s41598-022-18835-z

**Published:** 2022-08-26

**Authors:** Baoxin Ma, Junqiang Wang, Yehui Han, Chao Zhou, Ting Xu, Zhongcheng Qu, Lida Wang, Bo Ma, Ming Yuan, Lianxia Wang, Xinying Ding, Chunrong Qian

**Affiliations:** 1 Qiqihar Branch of Heilongjiang Academy of Agricultural Sciences, Qiqihar, China; 2 Institute of Tillage and Cultivation, Heilongjiang Academy of Agricultural Sciences, Harbin, China; 3 Animal Husbandry and Veterinary Branch of Heilongjiang Academy of Agricultural Sciences, Qiqihar, China

**Keywords:** Plant sciences, Plant development, Plant morphogenesis

## Abstract

Maize (*Zea mays* L.) is one of the most widely distributed and important crops in China. Maize ear differentiation plays an important role grain yield formation. However, it is unclear if ear and root morphophysiology status affects yield formation by altering ear differentiation and development under different nitrogen (N) conditions. The aim of this study is to understand how the ear differentiation and development are affected by ear and root morphophysiology traits, as affected by the N rate. The experiment consisted of two N rates: high nitrogen (180 kg ha^−1^), and low nitrogen (60 kg ha^−1^). Two N-efficient varieties (NEVs) and two N-inefficient varieties (NIVs) were grown in the field. The results showed higher nitrogen accumulation and grain yield in NEVs than in NIVs, which was mainly attributed to the increased N uptake by the larger root system under both N conditions. Under high N conditions, among ear differentiation-related traits, only FR was significantly positively correlated with grain yield, and NEVs ensure FR through higher N concentration and ZR content in ear at the fertilization stage. Under low N conditions, NEVs obtained higher FP, SR and FR through higher N concentration and IAA in ear at the early stage of ear differentiation, maintained lower AR and BTL by higher RA, R-ZR and E-ZR at the late stage of ear growth. These results suggest that NEVs have a more complex mechanism for obtaining higher grain yield under low N conditions than N sufficiency, and that phytohormones play an important role in this process.

## Introduction

It is expected that by 2050, the global demand for food will increase by 70%^1^. Nitrogen (N), a key limiting element for the growth of most crops, plays an important role in maximizing crop yields on a global scale, leading to a significant increase in the consumption of N fertilizers^2–4^. Global N fertilizer consumption has increased nine fold from 11 to 106 tonnes over the past 40 years (1960–2009), while grain yield has only increased by 164%^5^. During this period, maize production in China stagnated or even declined significantly^6,7^. Larger nitrogen input and lower nitrogen use efficiency will not only increase production costs and waste resources^8,9^, but also lead to soil acidification, decreased soil microbial activity, and adversely affect the environment. Sustainability presents challenges^10,11^. Therefore, future crop production should pay more attention to the efficient use of resources^12^. Further increases in grain yield must be achieved through increased nitrogen efficiency rather than higher nitrogen inputs^13,14^.

Developing the most suitable N fertilizer strategy for high-yielding crop systems is a critical step in achieving food security and environmental protection. Efficient use of N fertilizers is an effective strategy for both yield and profit. Studies have shown that crop rotation, no-tillage measures, the use of controlled-release nitrogen fertilizers, the deep application of urea, and the cultivation of NEVs (varieties with higher yields under both N conditions) can be achieved^15–18^, to overcome the depletion effects of excessive N application, thereby improving crop yield and agro-ecosystem sustainability. In general, genetic improvement of NEVs is an effective way to increase yield^19–23^.

Studies have also been conducted around N application levels, grain yield and N use efficiency, but the results vary widely due to differences in varieties, fertilizer levels, and climatic conditions^15,24^. Due to the continuous renewal of varieties, the production process puts forward higher requirements for nutrient input and efficiency improvement. The results of experiments on multiple corn varieties at multiple sites show that the selection of high-yield and high-efficient varieties can increase yield by 8–10%, while reducing N fertilizer input by 16–21%^9^. With the improvement of breeding technology, more maize varieties are used in production, but the nitrogen absorption and utilization among varieties show great differences. Nitrogen Use Efficiency (NUE) can be divided into Nitrogen Uptake Efficiency (NUpE) and Nitrogen Use Efficiency (NUtE). The former is mainly the nitrogen absorption capacity of plants, which is expressed as the nitrogen accumulation of plants; the latter is mainly the assimilation and redistribution capacity of plants to nitrogen, which is expressed as biomass or yield production capacity. Therefore, how to select varieties and combine their nitrogen utilization to meet the goals of high yield and resource efficiency is still a major problem to be solved by researchers.

Phytohormones are a class of organic substances produced by plants’ own metabolism, and participate in the regulation of various plant physiology and developmental processes. Some studies have shown that phytohormones play an important regulatory role in the process of seed growth and development^25^. The higher cytokinin (CTK) concentrations in seeds have been observed to be closely associated with rapid endosperm development during the early stages of seed development in cereals, peas, and soybeans^26–28^. Although CTKs are generally considered to be a class of hormones that regulate endosperm development, there is still insufficient evidence for the correlation between cytokinins and endosperm development. Auxin (IAA), gibberellin (GA) and abscisic acid (ABA) are also thought to be involved in the regulation of grain development^29^. Eeuwens et al.^30^ observed that the content of GA in endosperm cells was the highest during the elongation phase of pea pods. Some studies have shown that the content of GA in young panicles is higher at the flowering stage of rice and before flowering^31^. Lur et al.^32^ found that the concentration of IAA in endosperm cells increased rapidly on the tenth day after pollination of maize, which was consistent with the increase of deoxyribonucleic acid content in the nucleus. There are many research reports on the relationship between ABA content and seed growth and development, but the claim that it is involved in regulating the transport of assimilates to grains is still controversial^33–36^.

In this study, through a 2-year field experiment, four main summer maize varieties in Heilongjiang Province with similar growth periods were selected, and two fertilization levels of high (180 kg ha^−1^) and low (60 kg ha^−1^) N were set. To analyze the main reasons for the difference in yield of maize varieties with different N-efficiency under low and high nitrogen conditions. The root morphysiological characteristics of NEVs were systematically analyzed. Root and ear hormones were contained in ear differentiation and development stages, and their relationship with traits related to ear differentiation and development. This study improves our understanding of the roles of phytohormones in the regulation of morphological and physiological basis in N utilization and grain yield formation. This information could be used for research focused on improving N utilization and grain yield of maize.

## Materials and methods

### Plant materials and site description

Field experiments were conducted at the Qiqihar maize experiment base of heilongjiang academy of agricultural sciences in Heilongjiang Province, China (46° 52′ N, 123° 46′ E) during the rice growing season (May to October) in 2018 and 2019. The test area belongs to the mid-temperate continental monsoon climate, which is dry and windy in spring and warm and rainy in summer. The annual precipitation is 477 mm, and the frost-free period is about 130 days. The soil type was dark brown forest soil. The physicochemical properties of composite topsoil samples (0–20 cm) were determined, and the average values are shown in Table 1. Two N-efficient varieties (NEVs), Nengdan19 (ND19, N8924 × N7923) and Nengdan29 (ND29, N7923 × 1064), and two N-inefficient varieties (NIVs), Nengdan17 (ND17, N788411 × N1503) and Neng19022 (N19022, NH75121 × NY18), were used. The four maize varieties have similar growth periods. A crop rotation system was applied with continuous cropping of maize.

**Table 1:** The physicochemical property of composite topsoil samples (0–20 cm).

Year	Organic matter (g kg^−1^)	Total N content (g kg^−1^)	Rapidly available N (mg kg^−1^)	Rapidly available P (mg kg^−1^)	Rapidly available K (mg kg^−1^)	Value of pH
2018	23.12	0.91	67.52	23.21	146.8	7.23
2019	24.37	0.89	66.74	24.36	147.6	7.42

### Experimental design

The experiments were laid out in a complete randomized block design with three replicates. The N fertilizer rate was the main plot treatment, and the maize varieties formed the sub-plot treatments. Each plot was 30-m in length and 9.6-m in width with 60 cm row spacing. Maize was planted by hand and the planting density was 65,000 plant ha^−1^. Nitrogen rates were 180 kg ha^−1^ (high N conditions) and 60 kg ha^−1^ (low N conditions). Phosphorus (P_2_O_5_) rates were 90 kg ha^−1^ and potassium (K_2_O) rates were 120 kg ha^−1^. N, P, and K fertilizers were used urea, Ca(H_2_PO_4_)_2_ and K_2_SO_4_, respectively. All fertilizers were broadcasted before sowing. With the exception of the different N fertilizer rates, the other cultivation requirements were identical for all plots in both years. Chemicals were used to control diseases, pests and weeds to prevent yield loss during the experiment. Other field management is the same as conventional maize cultivation.

### Sampling and measurements

#### Grain yield and its components

Grain yield and its components were measured in 2018 and 2019. At R6 stage, two middle rows of plants (20-m length) per plot were harvested, excluding border plants. Grain yield was standardized to a moisture content of 0.14 g H_2_O g^−1^ and its components, i.e. grain number per ear (GNE) and thousand-kernel weight (TKW) were determined.

#### N accumulation and remobilization

In 2018 and 2019, five representative plants were taken from each plot in the R1 and R6 stages. The plants were divided into leaves, stems, cobs, bracts and grains. All parts were fixed at 105 °C for 30 min, and then dried at 80 °C until constant weight, weighed separately, and recorded the total mass of leaves, stems and plants. After crushing through a 100-mesh sieve, an 80 mg sample was weighed, and the nitrogen concentration of each part was measured using an elemental analyzer (EA1110, Thermo Electon SPA., Italy). The following indexes were calculated to investigate nitrogen accumulation and partitioning:

1. Straw N content at R1 (kg ha^−1^) = straw N concentration at R1 × straw dry weight at R1;
2. Straw N content at R6 (kg ha^−1^) = straw N concentration at R6 × straw dry weight at R6;
3. Grain N content at R6 (kg ha^−1^) = grain N concentration at R6 × grain dry weight at R6;
4. Nitrogen accumulation before silking (kg ha^−1^) = Straw N content at R1;
5. Nitrogen accumulation after silking (kg ha^−1^) = Straw and grain N content at R6 (kg ha^−1^) − Nitrogen accumulation before silking (kg ha^−1^);
6. Nitrogen remobilization (kg ha^−1^) = Straw N content at R1 − Straw N content at R6;
7. Cotibution of nitrogen remobilization (%) = NT/Grain N content at R6 × 100.

#### Ear traits and ear development

In 2018 and 2019, five representative ears per plot were sampled to determine row number per ear (RNE), grain number per row (GNR), ear length (EL) and barren tip length (BTL) at R6 stage. The RNE was the number of grains in the cross section in the middle of the ear. The GNR was the ratio of the number of grains per ear to the RNE. The EL was the axial distance from bottom to apex of ear. The BTL was the axial distance from the topmost grain to apex of ear.

At the R1 stage in 2018 and 2019, plants that entered the silking stage on the same day were marked. When maize silks withered, five representative ears per plot were sampled for determine the floret primordia (FP), silking number (SN), fertilized floret number (FFN) and aborted grain number (AGN). The FP was the number of all visible protrusions on the maize cob. After gently shaking the ear, the number of shedding filaments and unshedding filaments was the SN, where the number of shedding filaments was the FFN, and the AGN was the FFN minus the number of terminal grains. The following ratios were calculated to evaluate ear development:

1. Silking ratio (SR) = SN/FP × 100%;
2. Fertilized ratio (FR) = FFN/SN × 100%;
3. Aborted ratio (AR) = AGN/FFN × 100%.

#### Young ear biomass and nitrogen concentration during ear differentiation

In order to clarify the effect of nitrogen fertilizer on the ear differentiation of maize varieties, this study was based on the previous investigation of the silking period of the tested materials, and sampling was carried out around the silking period to determine the ear nitrogen concentration, ear dry weight, root morphysiological characteristics, ear and root phytohormone content. The stages were S1 (R1 − 14d), S2 (R1 − 7d), S3 (R1), S4 (R1 + 7d) and S5 (R1 + 14d). Five representative ears per plot were sampled from S1 to S5 stages. All ears were fixed at 105 °C for 30 min, and then dried at 80 °C until constant weight, weighed separately, and recorded the total mass of ears. After crushing through a 100-mesh sieve, an 80 mg sample was weighed, and the nitrogen concentration of each part was measured using an elemental analyzer (EA1110, Thermo Electon SPA., Italy).

#### Root morphological and physiological traits during ear differentiation

Root samples were taken from S1 to S5, and the whole plant was excavated for sampling. Five representative plants with no marginal effect were excavated from each plot. The sampling depth was 60 cm, with the plant as the center, and the sampling area was 0.15 m^2^ (length 0.6 m, width 0.25 m). The excavated roots were washed with clean water and packed into ziplock bags, brought back to the laboratory, and scanned and analyzed with Win RHIZO Pro 2007d (Regent Instruments Inc., Quebec, Canada) to obtain the total root length. The treated roots were dried in an oven at 80 °C to constant weight, and the dry weight of the roots was recorded. The root activity (RA) was measured using the method described by Ramasamy et al.^37^.

#### Ear and root phytohormones content during ear differentiation

The ear and root samples were freeze dried with liquid N and stored at − 80 °C refrigerator for phytohormone analysis. The IAA, ABA, GA and ZR were extracted by enzyme-linked immunosorbent assay (ELIAS) with reference to the operation guide of China Agricultural University ELIAS kit^38,39^. The IAA, ABA, GA and ZR content were determined using a Multiskan™ FC Microplate Photometer (Thermo Fisher Scientific (China) Co., Ltd., Shanghai, China).

### Statistical analysis

For experimental variables, one-way of variance (ANOVA) was applied to assess differences among treatments with SPSS 22.0 software (SPSS Inc., Chicago, IL, USA). Significant differences between treatments are indicated by different letters at P < 0.05 level according to Fisher’s LSD. Graphs were drawn with Origin 2018 software (OriginLab, Northampton, MA, USA) R software (Available online: http://www.r-project.org/) and Adobe Illustrator CS6 (Adobe Systems Inc., CA, USA).

### Statement

The authors ensure that all maize seeds used in this study originated from Qiqihar Branch of Heilongjiang Academy of Agricultural Sciences in Heilongjiang Province, China. The legality of these seeds complies with the IUCN Policy Statement on Research Involving Species at Risk of Extinction and the Convention on the Trade in Endangered Species of Wild Fauna and Flora. The maize seeds collected in the study are all cultivated maize in China rather than endangered and wild species. These varieties have passed the legal variety certification procedures in China and are licensed for production, planting, and market operations. The authors declare that the cultivation of plants and carrying out study in the Qiqihar maize experiment base of Heilongjiang academy of agricultural sciences complies with all relevant institutional, national and international guidelines and treaties.

## Results

### Post-silking N accumulation and remobilization

As shown in Table 2, the nitrogen accumulation before silking and after silking under low nitrogen condition was significantly lower than that under high nitrogen condition. The nitrogen accumulation before silking and after silking in NEVs were significantly higher than that in NIVs under both N conditions. The nitrogen remobilization and contribution of nitrogen remobilization under low nitrogen condition was significantly higher than that under high nitrogen condition. The nitrogen remobilization and contribution of nitrogen remobilization in NEVs were significantly lower than that in NIVs under both N conditions.

**Table 2:** Post-silking nitrogen accumulation and remobilization of the four varieties under different nitrogen rate.

Year	Treatment	Varieties	Nitrogen accumulation before ailking (kg ha^−1^)	Nitrogen accumulation after ailking (kg ha^−1^)	Nitrogen remobilization (kg ha^−1^)	Contribution of nitrogen remobilization (%)
2018	HN	ND19	129.44 ± 2.88a	72.53 ± 1.53a	31.96 ± 1.5b	30.57 ± 1.74d
		ND29	124.19 ± 2.23b	70.49 ± 1.52a	35.03 ± 1.76b	33.19 ± 1.35c
		ND17	117.16 ± 1.28c	63.3 ± 1.26b	41.49 ± 1.73a	39.58 ± 1.47b
		N19022	113.89 ± 2.52c	58.72 ± 1.29c	43.01 ± 2.53a	42.26 ± 1.99a
	LN	ND19	103.25 ± 4.65a	22.55 ± 1.03a	46.66 ± 3.27ab	67.35 ± 1.87b
		ND29	95.32 ± 2.31b	21.86 ± 0.95a	43.2 ± 1.93b	66.36 ± 1.96b
		ND17	86.57 ± 1.75c	18.53 ± 0.49b	46.57 ± 1.49ab	71.52 ± 0.97a
		N19022	87.37 ± 2.48c	17.7 ± 0.84b	49.52 ± 1.94a	73.66 ± 1.04a
2019	HN	ND19	142.31 ± 1.61a	75.16 ± 0.9a	40.76 ± 1.49b	35.15 ± 0.90b
		ND29	133.15 ± 1.26b	76.13 ± 1.64a	40.55 ± 2.98b	34.71 ± 2.17b
		ND17	126.29 ± 1.77c	63.52 ± 1.56b	50.37 ± 2.85a	44.19 ± 2.01a
		N19022	124.01 ± 1.08c	64.67 ± 1.31b	48.96 ± 1.23a	43.08 ± 0.89a
	LN	ND19	112.3 ± 1.69a	23.14 ± 1.13a	53.45 ± 1.10b	69.79 ± 1.18c
		ND29	107.68 ± 1.56a	22.65 ± 0.87a	53.36 ± 1.58b	70.19 ± 1.48c
		ND17	95.43 ± 3.1b	19.17 ± 0.44b	53.93 ± 2.62b	73.73 ± 1.47b
		N19022	99.41 ± 3.59b	18.41 ± 0.81b	59.55 ± 1.44a	76.37 ± 1.23a
F-value	Y		235.75**	45.76**	180.06**	44.88**
	N		1607.68**	22,415.2**	245.5**	6150.33**
	V		111.83**	191.99**	37.19**	96.72**
	Y** × **V		0.65	21.24**	1.2	0.01
	Y** × **V		0.94	4.76**	0.02	0.89
	N** × **V		2.29	39.99**	10.98**	6.62**
	Y × N × V		2.44	4.32**	2.25	2.21

*HN* high nitrogen conditions, *LN* low nitrogen conditions.

**F values significance at 0.05 and 0.01 probability levels, respectively.

Different letters represent significant differences among varieties.

### Grain yield and its components

**Figure 1:**
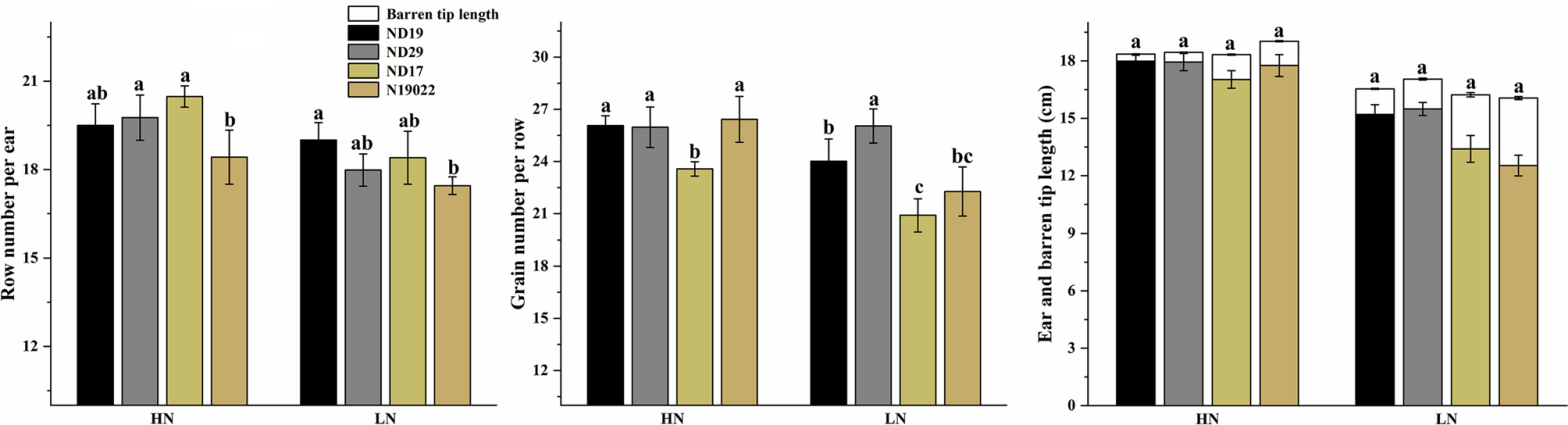
Row number per ear, grain number per row, ear and barren tip length of the four varieties under different nitrogen rate. Result is the average from 2018 to 2019. Error bars are given as S.E. *HN* high nitrogen conditions, *LN* low nitrogen conditions. The different small letters above the column indicate significant difference among cultivars in the same N condition during the same growth stage at *P* < 0.05.

The grain yield (GY) under high nitrogen conditions was significantly higher than that under low nitrogen conditions in all varieties. The GY was significantly higher for NEVs than for NIVs under both N conditions (except for under high nitrogen conditions in 2019) (Table 3). As shown in Table 3, compared with high nitrogen conditions, the grain number per ear and thousand-kernel weight were significantly decreased under low nitrogen conditions, the grain number per ear in NEVs and NIVs were reduced 8.63–15.51% and 19.67–20.50%, respectively. The grain number per ear were significantly higher for NEVs than for NIVs under both N conditions. Compared with high nitrogen conditions, the thousand-kernel weight in NEVs and NIVs were reduced 5.97–11.59% and 9.44–11.62%, respectively. This result indicates that the grain number per ear is the main reason for the difference in yield of different nitrogen-efficiency varieties under both nitrogen conditions. As shown in Fig. 1, compared with high nitrogen conditions, the row number per ear, grain number per row, and ear length were significantly decreased, and the barren tip length were significantly increased under low nitrogen conditions. The ear length of NEVs were significantly higher than that of NIVs under low nitrogen conditions, the barren tip length of NEVs were significantly lower than that of NIVs under both nitrogen conditions.

**Table 3:** Grain yield and yield components of the four varieties under different nitrogen rate.

Year	Treatment	Varieties	Grain number per ear	Thousand-kernel weight (g)	Grain yield (t ha^−1^)
2018	HN	ND19	487.03 ± 9.12a	326.65 ± 7.7a	10.33 ± 0.14a
		ND29	507.59 ± 17.78a	303.01 ± 7.04b	10.96 ± 0.27a
		ND17	469.34 ± 4.44b	329.34 ± 9.39a	9.86 ± 0.3b
		N19022	480.73 ± 6.73ab	324.69 ± 7.72a	9.52 ± 0.13b
	LN	ND19	432.40 ± 12.09a	297.11 ± 6.46a	8.44 ± 0.19a
		ND29	428.87 ± 10a	280.9 ± 5.6b	8.24 ± 0.25a
		ND17	377.01 ± 7.3b	297.99 ± 7.35a	7.12 ± 0.23b
		N19022	385.13 ± 7.84b	289.54 ± 8.28ab	7.23 ± 0.28b
2019	HN	ND19	507.65 ± 10.42a	362.16 ± 14.16a	11.45 ± 0.21b
		ND29	511.92 ± 4.23a	333.78 ± 7.48b	12.04 ± 0.27a
		ND17	482.69 ± 8.39b	361 ± 7.32a	11.03 ± 0.56b
		N19022	485.26 ± 13.88b	358.01 ± 11.06a	10.48 ± 0.29c
	LN	ND19	455.00 ± 10.96a	320.19 ± 7.34a	9.17 ± 0.29a
		ND29	467.72 ± 7.98a	313.87 ± 11.55a	8.98 ± 0.3a
		ND17	383.73 ± 2.09b	326.92 ± 12.4a	7.82 ± 0.25b
		N19022	388.32 ± 15.6b	316.40 ± 10.86a	8.02 ± 0.19b
F-value	Y		23.67**	120.90**	134.32**
	N		684.44**	133.78**	1080.92**
	V		74.69**	11.54**	63.25**
	Y** × **V		1.48	0.77	4.79*
	Y** × **V		2.26	0.04	0.03
	N** × **V		14.58**	1.92	7.30**
	Y × N × V		2.51	0.31	0.16

*HN* high nitrogen conditions, *LN* low nitrogen conditions.

* and **F values significance at 0.05 and 0.01 probability levels, respectively.

Different letters represent significant differences among varieties.

### Floret and grain development

As shown in Table 4, the floret and grain development were affected by varieties and nitrogen rates. Compared with high nitrogen conditions, the floret primordia were significantly decreased under low nitrogen conditions, the floret primordia in NEVs and NIVs were reduced 2.22–4.15% and 5.09–10.33%, respectively. The silking ratio and fertilized ratio of NEVs were significantly higher than that of NIVs under both nitrogen conditions, and nitrogen availability has less effect on silking ratio and fertilized ratio. Compared with high nitrogen conditions, the abortion ratio was significantly increased under low nitrogen conditions, the abortion ratio in NEVs and NIVs were increased 54.40–87.41% and 92.13–106.31%, respectively. In addition, the abortion ratio of NEVs were significantly lower than NIVs under low nitrogen conditions.

**Table 4:** Floret growth and development of the four varieties under different nitrogen rate.

Year	Treatment	Varieties	Floret primordia	Silking ratio (%)	Fertilized ratio (%)	Abortion ratio (%)
2018	HN	ND19	741.47 ± 10.08b	90.84 ± 0.66a	82.43 ± 0.38a	12.28 ± 0.16a
		ND29	771.41 ± 22.84a	90.09 ± 0.43a	81.94 ± 0.66a	10.88 ± 0.36b
		ND17	782.58 ± 13.54a	87.49 ± 0.86b	77.88 ± 1.65b	11.94 ± 0.35a
		N19022	785.09 ± 3.13a	87.44 ± 0.67b	79.82 ± 0.95b	12.27 ± 0.47a
	LN	ND19	710.67 ± 7.08b	88.42 ± 0.75a	84.90 ± 1.64a	18.96 ± 0.53c
		ND29	739.96 ± 12.01a	88.57 ± 1.07a	82.20 ± 0.58a	20.39 ± 0.46b
		ND17	701.75 ± 9.27b	86.40 ± 0.48b	80.69 ± 0.68b	22.94 ± 1.42a
		N19022	739.88 ± 8.75a	85.98 ± 0.94b	79.58 ± 1.95b	23.89 ± 0.94a
2019	HN	ND19	780.46 ± 9.02a	90.71 ± 0.95a	82.08 ± 1.76a	12.62 ± 0.36a
		ND29	797.49 ± 7.44a	89.02 ± 1.04a	82.79 ± 1.04a	12.88 ± 0.36a
		ND17	775.84 ± 4.41b	87.90 ± 0.31b	80.04 ± 0.56b	11.57 ± 1.42b
		N19022	760.41 ± 9.58b	87.80 ± 1.01b	80.40 ± 1.39b	11.86 ± 0.26a
	LN	ND19	763.12 ± 10.01a	88.51 ± 0.49a	83.61 ± 0.71b	19.44 ± 0.84b
		ND29	772.89 ± 19.71a	90.18 ± 0.71a	84.77 ± 1.13a	20.81 ± 0.72b
		ND17	713.39 ± 7.13b	87.45 ± 0.86b	80.81 ± 0.72d	23.87 ± 0.72a
		N19022	721.72 ± 11.51b	85.38 ± 0.69c	82.46 ± 0.39c	23.61 ± 2.09a
F-value	Y		18.67**	0.89*	14.41**	4.24*
	N		161.89**	32.84**	9.71**	1477.59**
	V		12.99**	43.99**	20.44**	19.45**
	Y × V		3.02	2.07	0.51	0.27
	Y × V		20.72**	0.7	5.81**	0.91
	N × V		10.97**	4.83**	2.87*	21.04**
	Y × N × V		0.19	2.79*	1.3	1.59

*HN* high nitrogen conditions, *LN* low nitrogen conditions.

* and **F values significance at 0.05 and 0.01 probability levels, respectively.

Different letters represent significant differences among varieties.

### The relationships among grain yield and the ear and grain development

**Figure 2:**
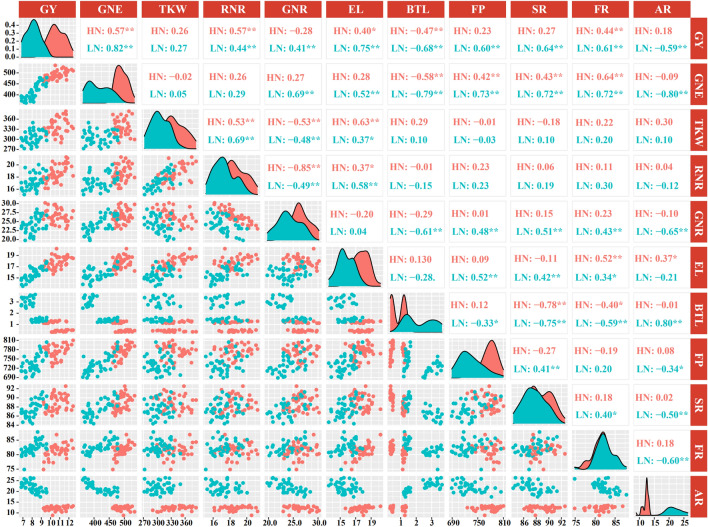
The relationship between Grain yield and its components and ear differentiation traits under both N conditions. The * and **indicate that at the level of 0.05 and 0.01, respectively. *GN* grain yield, *GNE* grain number per ear, *TKW* thousand-kernel weight, *RNR* row number per ear, *GNR* grain number per row, *EL* ear length, *BTL* barren tip length, *FP* floret primordia, *SR* silking ratio, *FR* fertilized ratio, *AR* aborted ratio.

The correlations between GY and the ear and grain development were shown in Fig. 2. Under low N conditions, GNE, RNE, GNR, EL, FP, SR and FR were strongly and positively, and BTL and AR was strongly and negatively correlated with GY. Under high N conditions, GNE, RNR, GNR and FR were strongly and positively, and BRL was strongly and negatively correlated with GY. Under low N conditions, GNR, EL, FP, SR and FR were strongly and positively, and BTL, and AR were strongly and negatively correlated with GNE. Under high N conditions, FP, SR and FR was strongly and positively, BTL was strongly and negatively correlated with GNE.

### Young ear biomass and nitrogen concentration during ear differentiation

**Figure 3:**
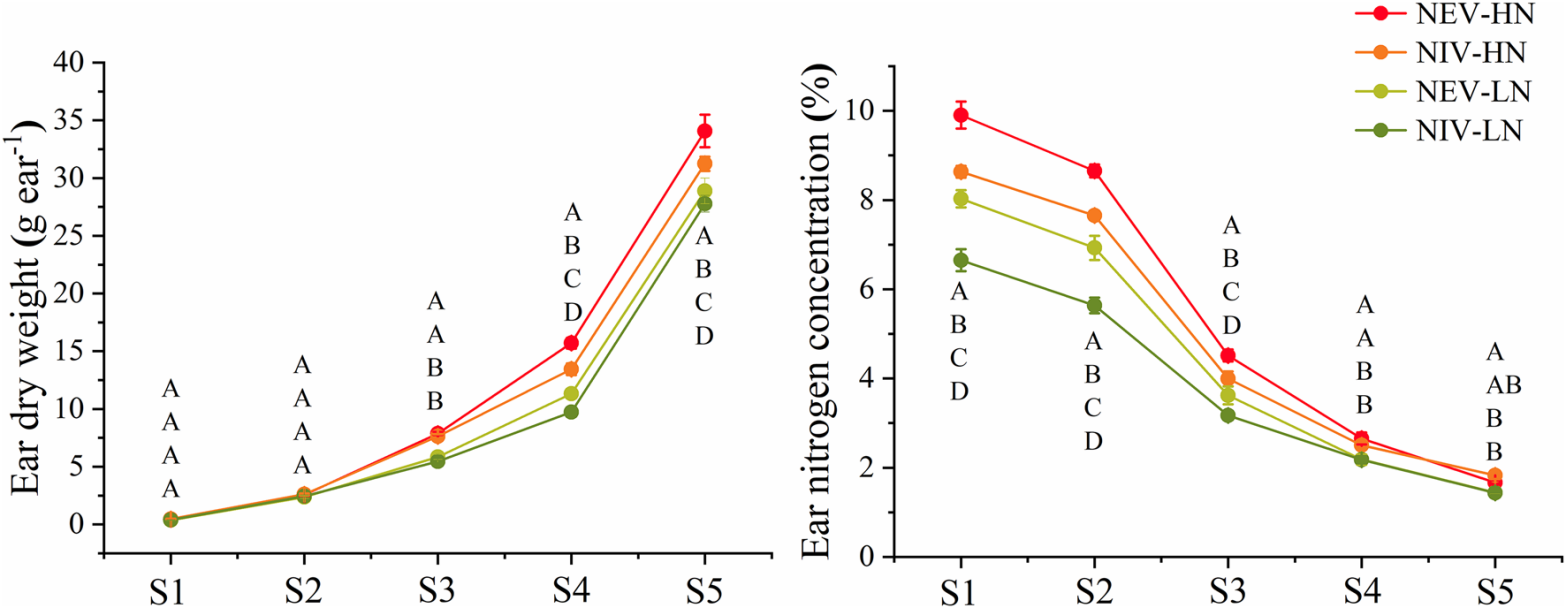
Dynamics of ear dry weight and nitrogen concentration during critical ear differentiation stages. Each point represents a replicate deriving from 2018 to 2019. *HN* high nitrogen conditions, *LN* low nitrogen conditions. The letter as indicates the difference between varieties at P < 0.05. From top to bottom, represent NEV-HN, NIV-HN, NEV-LN and NIV-LN.

As shown in Fig. 3, during the main period of ear development, the responses of different nitrogen-efficiency varieties to nitrogen fertilizer were quite different. With the development of ear, the biomass of young ear increased and the nitrogen concentration decreased. There were significant differences in ear nitrogen concentration among different nitrogen rate and nitrogen efficiency varieties during S1 to S3 period, the nitrogen concentration under high nitrogen condition were significantly higher than that under low nitrogen condition, and the nitrogen concentration of NEVs were significantly higher than NIVs under both nitrogen conditions. During the period from S3 to S5, there were significant differences in ear biomass among different nitrogen fertilizer treatments and nitrogen efficiency varieties. The ear dry matter mass under high nitrogen conditions were significantly higher than that under low nitrogen conditions, and the ear biomass mass of NEVs were significantly higher than NIVs under both nitrogen conditions.

### The morphological and physiological characteristics of the root during spike differentiation

**Figure 4:**
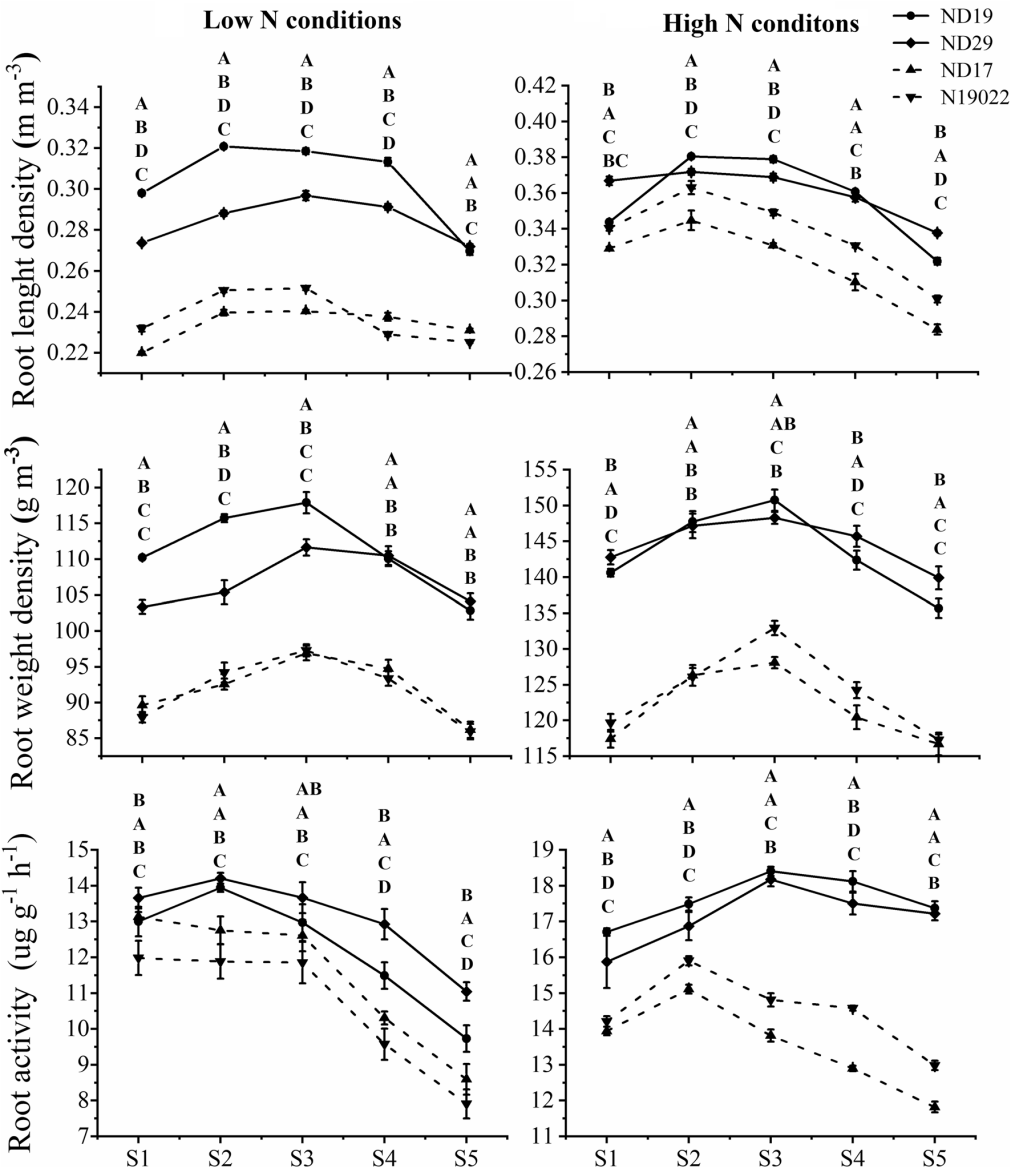
Dynamics of root morphological and physiological traits during critical ear differentiation stages. Each point represents a replicate deriving from 2018 to 2019. The letter as indicates the difference between varieties at P < 0.05, from top to bottom, represent ND19, ND29, ND17 and N19022.

The RLD of all varieties showed a trend of increasing first and then decreasing from the S1 to S5 stage, the maximum value occurs at the S2 or S3 stage under both N conditions (Fig. 4). The RLD were significantly higher for NEVs than for NIVs from the S1 to S5 under both N conditions. The RWD of all varieties showed a trend of increasing first and then decreasing from the S1 to S5 stage, the maximum value occurs at the S3 stage under both N conditions. The RWD were significantly higher for NEVs than for NIVs from the S1 to S5 under both N conditions. The RA of all varieties was not significantly different from S1 to S3 stage, and gradually decreased from S3 to S5 under low nitrogen conditions. While the RA of all varieties increased and then decreased from S1 to S5 stage, the maximum value occurs of NEVs at the S3 stage, the maximum value occurs of NIVs at the S2 stage under high nitrogen conditions. Overall, NEVs can expand root distribution in the growing environment by promoting root elongation and maintain higher root activity.

### The phytohormone content in young ear during ear differentiation

**Figure 5:**
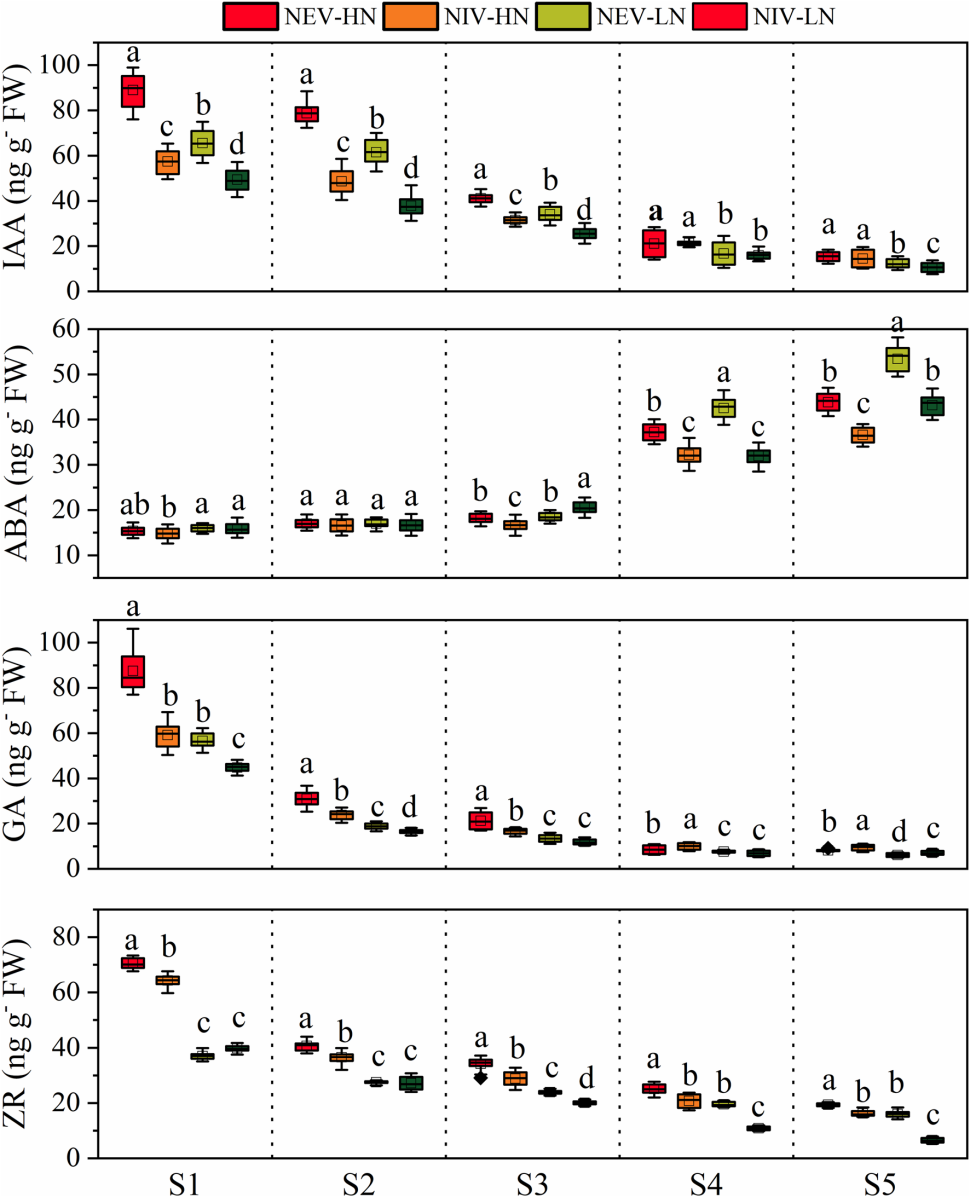
The IA, ABA, GA and ZR of ear during critical ear differentiation stages. Result is the average from 2018 to 2019. *NEV-HN* NEVs under high nitrogen conditions, *NIV-HN* NIVs under high nitrogen conditions, *NEV-LN* NEVs under low nitrogen conditions, *NIV-LN* NIVs under low nitrogen conditions. The different small letters above the box indicate significant difference among the same ear differentiation stage at *P* < 0.05.

The young ear phytohormones content are presented in Fig. 5. The IAA content in the ear of all varieties decreased from the S1 to S5 stage under both N conditions, and was significantly higher in NEVs than in NIVs from the S1 to S3 stage under both N conditions. The ABA content in the ear of all varieties increased from the S1 to S5 stage under both N conditions, was significantly higher in NEVs than in NIVs at the S4 and S5 stage under both N conditions. The GA content in the ear of all varieties decreased from the S1 to S5 stage under both N conditions, and was significantly higher in NEVs than in NIVs from the S1and S2 stage under both N conditions. The ZR content in the ear of all varieties decreased from the S1 to S5 stage under both N conditions, and was significantly higher in NEVs than in NIVs from the S2 to S5 stage under both N conditions.

### The phytohormone content in root during ear differentiation

**Figure 6:**
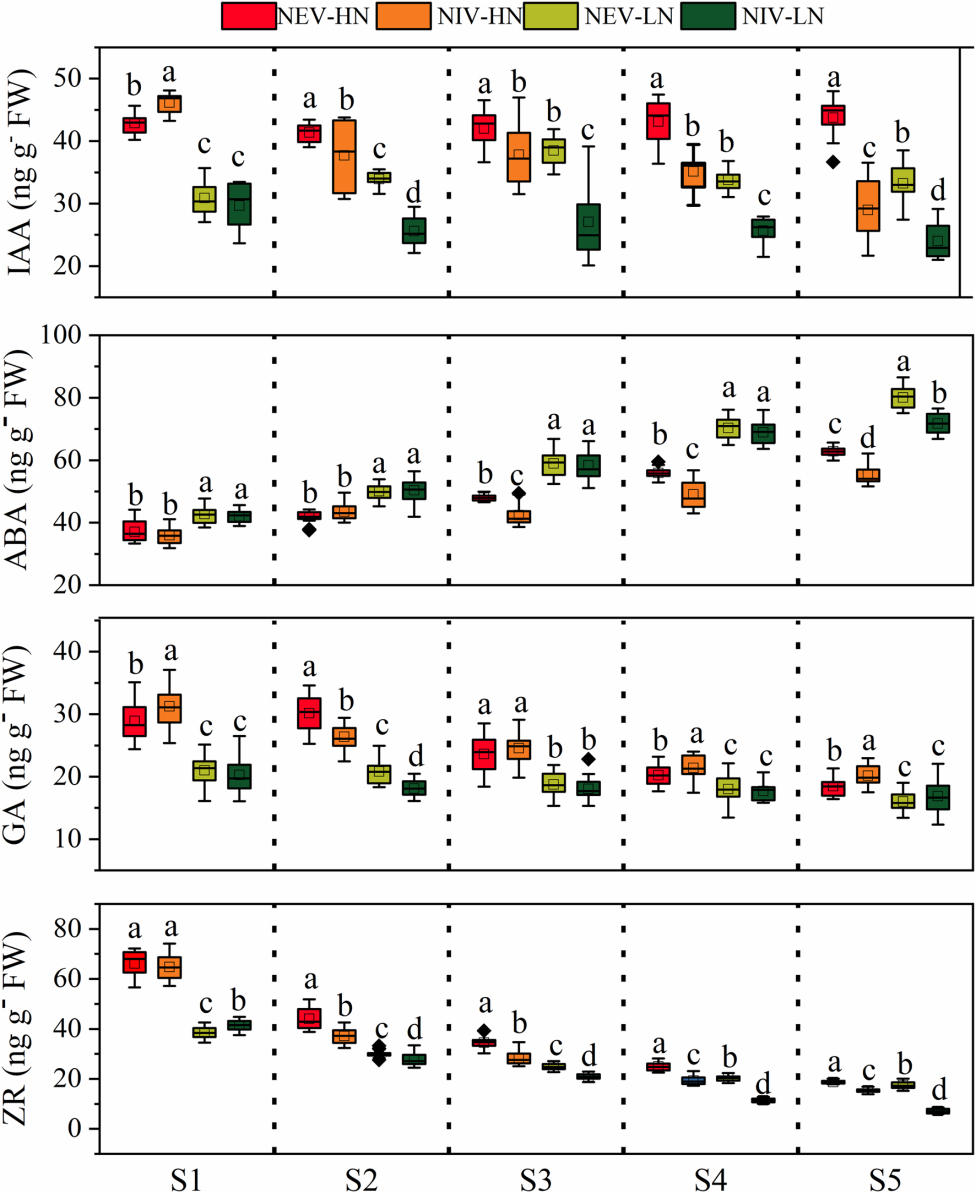
The IA, ABA, GA and ZR of root during critical ear differentiation stages. Result is the average from 2018 to 2019. *NEV-HN* NEVs under high nitrogen conditions, *NIV-HN* NIVs under high nitrogen conditions, *NEV-LN* NEVs under low nitrogen conditions, *NIV-LN* NIVs under low nitrogen conditions. The different small letters above the box indicate significant difference among the same ear differentiation stage at *P* < 0.05.

The root phytohormones content are presented in Fig. 6. There was no significant difference in IAA content in roots at different ear development stages under both N conditions, and was significantly higher in NEVs than in NIVs from the S2 to S5 stage under both N conditions. The ABA content in the root of all varieties increased from the S1 to S5 stage under both N conditions, and was significantly higher in NEVs than in NIVs from the S3 to S5 stage under high N conditions, and at the S5 stage under low N conditions. The GA content in the root of all varieties decreased from the S1 to S5 stage under high N conditions, and was significantly higher in NEVs than in NIVs at the S1, S4 and S5 stage under high N conditions. The ZR content in the root of all varieties decreased from the S1 to S5 stage under both N conditions, and was was significantly higher in NEVs than in NIVs from the S2 to S5 stage under both N conditions.

### Correlations between ear and grain development, and leaf and root phytohormones

**Figure 7:**
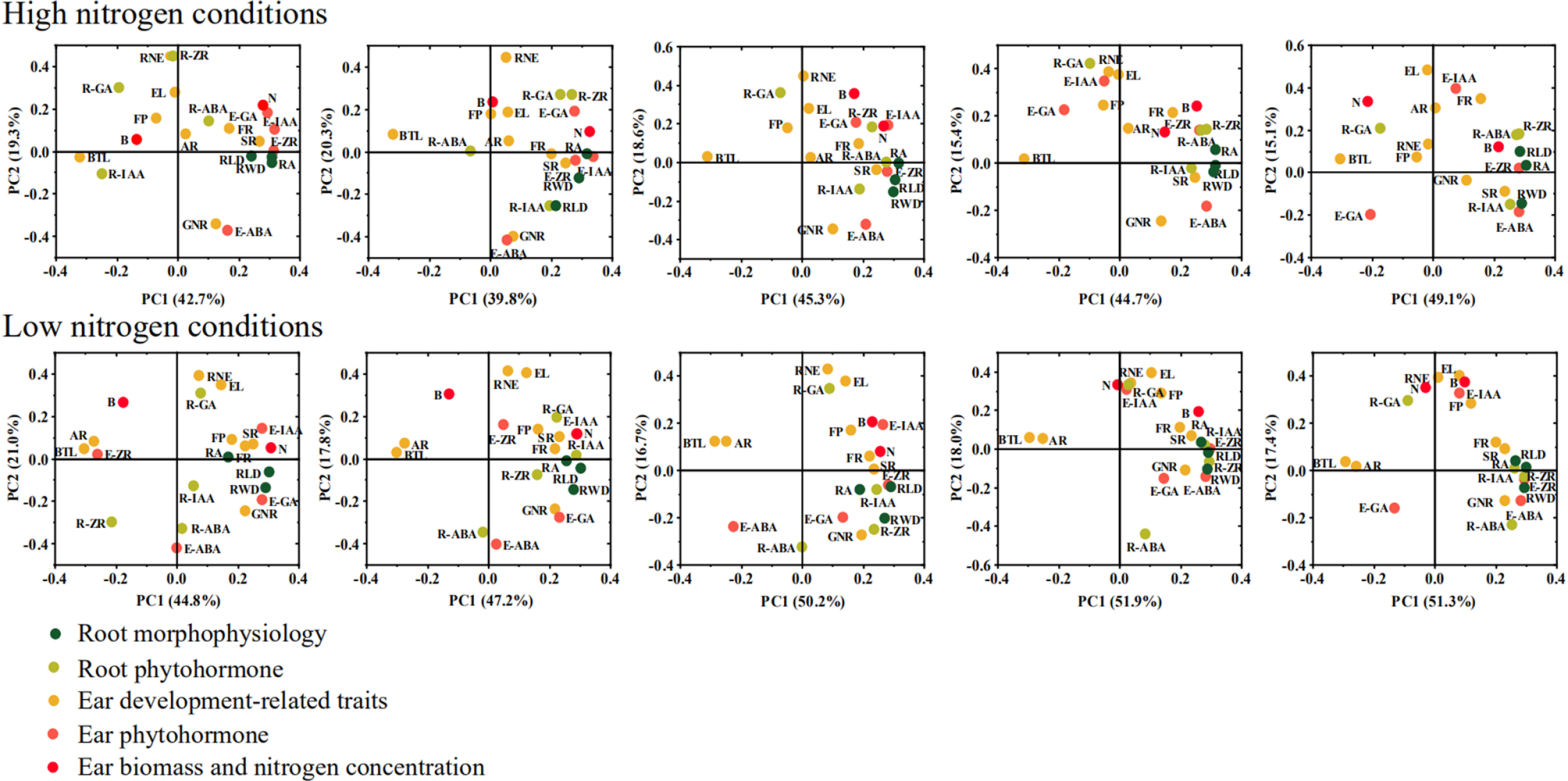
Principal component analysis (PCA) of ear differentiation and development traits, ear and root phytohormones, root morphological and physiological traits determined on maize varieties during critical ear differentiation stages.

The results of correlation analysis (Fig. 2) and principal component analysis (Fig. 7) showed that FR was the main factor causing the GY and GNE difference under high nitrogen conditions, while FP, SR, FR and AR were the main factors affecting GY and GNE under low nitrogen conditions. Under high N conditions, the N concentration of ear and E-IAA content were significantly positively correlated with FR from the S1 to S4 stage, while under low N conditions, the N concentration of ear and E-IAA content were significantly positively correlated with FR and SR Correlated from S1 to S3 stages, AR was significantly negatively correlated with RA, R-ZR and E-ZR from S3 to S5.

## Discussion

There is great genetic variability in both nitrogen uptake efficiency and nitrogen physiological use efficiency in maize. In many cases, maize varieties that perform best under high nitrogen fertilizer inputs do not necessarily perform well when nitrogen supplies are reduced^40^, which is related to nitrogen management strategies and environmental factors^41^. Nitrogen uptake and nitrogen utilization efficiency of different maize varieties tend to be opposite at different nitrogen supply levels. Due to the complex and diverse indicators related to nitrogen absorption and utilization efficiency, and the grain yield directly reflects the economic value of maize. Therefore, it is simple and feasible to use maize grain yield under different nitrogen supply levels as a criterion for evaluating nitrogen-efficient maize varieties. In this study, the grain yield of maize under high and low N conditions was used to evaluate nitrogen-efficient corn varieties. Two NEVs, ND19 and ND29, and two NIVs, ND17 and N19022, were obtained. The results of nitrogen accumulation and transport showed that, compared with the NIVs, the NEVs had higher nitrogen uptake capacity, while the nitrogen remobilization was relatively lower in NEVs. The lower nitrogen remobilization is mainly due to the large amount of nitrogen accumulation in the NEVs, which does not require a large amount of leaf nitrogen to be transferred, while the higher leaf nitrogen accumulation is beneficial to maintain the carbon assimilation and transport capacity of leaves in the later stages of growth. This result suggests that higher nitrogen uptake is beneficial for higher maize yields regardless of low or high N supply.

The grain yield decreased with the reduction of nitrogen application rate, and the grain number per ear is an important factor reflecting the storage capacity of the ear, which is the main reason for the difference in grain yield of maize varieties with different nitrogen efficiency. Compared with NEVs, low nitrogen had a greater effect on the grain number per ear of NIVs. The formation time of grain number per ear is from the early stage of spinning (the first visible filament is exposed) to about two weeks after spinning. The ear differentiation is the key to determine grain number per ear^42^. The ear differentiation stage is from the formation of florets in the early stage of silking to about two weeks after silking^43^. The relevant indicators of grain number per ear formation period are regulated by genetic and environmental factors, including the floret primordia, silking ratio, fertilized ratio and abortion ratio. Previous studies have shown that nutrient stress has no significant effect on floret primordia^44–46^, while the present study found that low nitrogen stress significantly decreased floret primordia. Nitrogen deficiency can trigger asynchronous pollination^47^, thus affecting the number of fertilized florets, or it may also reduce the supply of NSCs at the onset of grain filling, which may in turn affect grain abortion (especially in the apical ones grains) have an effect. Previous studies have shown that filament growth dynamics under genotypic differences may be related to the ability of varieties to maintain pollination, which plays a crucial role in determining the grain number per ear^48,49^. Low nitrogen stress significantly increased aborted grains and unpollinated filaments, resulting in a decrease in grain number. Rossini et al.^50^ pointed out that the grain number per ear due to N-deficient grain abortion can be reduced by up to 41%, and it is also reduced by 20% under sufficient N conditions. In the present study, low N significantly decreased silking ratio, fertilized ratio and increased abortion ratio compared with high N conditions. Under the low N conditions, the genotypic differences in the grains number per ear were related to floret differentiation, the lower proportion of grain abortion in unfertilized and fertilized florets, and NEVs were less affected by low N conditions.

In order to clarify the physiological and biochemical basis of the response of grain number formation-related traits to nitrogen supply levels, this study measured the ear nitrogen concentration and phytohormone content, root morphophysiological characteristics and root phytohormone content at the main stages of young ear development. There are few studies on the relationship between the shoots and roots of different varieties under field conditions, and most of the previous related studies were carried out under potted conditions. The morphology of the underground part is closely related to the growth status of the above-ground part of the plant, and plants with larger root biomass are usually more conducive to obtaining soil nutrients^51,52^, corresponding to The aboveground biomass is also larger. In the present study, the RLD, RWD and RA of NEVs were significantly larger than those of NIVs under both nitrogen conditions. The RLD, RWD and RA were significantly reduced by low N, and NEVs were relatively less affected. In this study, it is believed that the excellent root index of NEVs ensures the high ear nitrogen concentration in the early stage of ear development. These results indicated that NEVs can coordinate root morphophysiological to obtain more N and produce higher grains number per ear under both N conditions.

Phytohormones play an important role in synergistically regulating the growth and development of rice organs, nutrient absorption, carbon and N assimilation, transport and distribution as well as inducing defensive adaptation to stress^53^. IAA is the earliest phytohormone discovered, and its content is very low in plants, but it plays an important role in crop organogenesis and morphogenesis, tissue differentiation tropism and apical dominance^54,55^. Higher N concentration in differentiated organs is beneficial to promote IAA synthesis to promote organ differentiation^56,57^. ABA is known as the senescence hormone, which can promote the senescence of plants, but the role of abscisic acid in the senescence process is contradictory. Some studies have shown that ABA can coordinate the senescence process to ensure grain yield in the later stages of crop growth^58–60^. ZR is a plant endogenous hormone, which belongs to cytokinin, which can promote cell division and is mainly synthesized in roots^61^, the ZR content of ear was significantly correlated with the ZR content of root in the middle and late stages of ear development under both N conditions. In this study, compare with high N conditions, the IAA, GA, and ZR levels increased and ABA levels decreased under low N conditions, which is consistent with previous studies^62,63^. The results of the PCA showed that the IAA content of the ear was significantly correlated with the N concentration in the ear, especially under low nitrogen conditions, they were significantly positively correlated with the FP, SR and FR at the floret differentiation stage (S1 to S3). Furthermore, under low N conditions, the AR and BTL was significantly negatively correlated with RA, R-ZR and E-ZR from S3 to S5 stage. These results suggest that NEVs can coordinate the phytohormone content of ears and roots at different stages of ear differentiation, resulting in higher grain numbers per ear under low N conditions.

## Conclusions

Compared with NIVs, NEVs showed a stronger tolerance to low N stress, and a higher yield potential under high N conditions. Under both N conditions, compared with NIVs, NEVs showed greater RLD, RWD, and RA in the ear growth stages, which brought higher ear N concentration and laid a favorable nutritional foundation for ear differentiation and growth. Under high N conditions, among ear differentiation-related traits, only FR was significantly positively correlated with grain yield, and NEVs ensure FR through higher N concentration and ZR content in ear at the fertilization stage. Under low N conditions, NEVs obtained higher FP, SR and FR through higher N concentration and IAA in ear at the early stage of ear differentiation, maintained lower AR and BTL by higher RA, R-ZR and E-ZR at the late stage of ear growth. These results suggest that NEHs have a more complex mechanism for obtaining higher grain yield under low N conditions than N sufficiency, and that phytohormones play an important role in this process. Further research is needed to understand the mechanism of exogenous phytohormones in the regulation of ear differentiation and development, and to improve the grain yield in maize under different N supply levels.

## Data Availability

The datasets used and/or analysed during the current study available from the corresponding author on reasonable request (C.Q., qianjianyi318@163.com).
